# Aortic Arch Calcification and the Risk of Cancer: A Population-Based Cohort Study

**DOI:** 10.3389/fonc.2020.01700

**Published:** 2020-09-11

**Authors:** Janine E. van der Toorn, Kimberly D. van der Willik, Rikje Ruiter, Meike W. Vernooij, Bruno H.Ch. Stricker, Sanne B. Schagen, M. Arfan Ikram, Maryam Kavousi, Daniel Bos

**Affiliations:** ^1^Department of Epidemiology, Erasmus MC - University Medical Center, Rotterdam, Netherlands; ^2^Department of Radiology and Nuclear Medicine, Erasmus MC - University Medical Center, Rotterdam, Netherlands; ^3^Department of Psychosocial Research and Epidemiology, Netherlands Cancer Institute, Amsterdam, Netherlands; ^4^Brain and Cognition, Department of Psychology, University of Amsterdam, Amsterdam, Netherlands; ^5^Department of Clinical Epidemiology, Harvard T.H. Chan School of Public Health, Boston, MA, United States

**Keywords:** atherosclerosis, calcification, cancer, cohort study, survival

## Abstract

**Background:** Atherosclerosis and cancer share multiple disease pathways. Yet, it is unclear if atherosclerosis is associated with a subsequent higher cancer risk. We determined the association of atherosclerotic calcification in the aortic arch, as proxy for systemic atherosclerosis, with the risk of cancer.

**Methods:** Between 2003 and 2006, 2,404 participants (mean age: 69.5 years, 52.5% women) from the prospective population-based Rotterdam Study underwent computed tomography to quantify calcification in the aortic arch. Participants were followed for the onset of cancer, death, loss to follow-up, or January 1st, 2015, whichever came first. We computed sex-specific tertiles of aortic arch calcification volumes. Next, we examined the association between the volume and severity (i.e., tertiles) of aortic arch calcification and the risk of cancer using Cox proportional hazard models.

**Results:** During a median (interquartile range) follow-up of 9.6 years (8.9–10.5), 348 participants were diagnosed with cancer. Participants with the greatest severity of aortic arch calcification had a higher risk of cancer [hazard ratio for the third tertile compared to the first tertile of aortic arch calcification volume in the total population is 1.39 (95% CI = 1.04–1.86)].

**Conclusions:** Individuals with the most severe aortic arch calcification had a higher risk of cancer. While this could reflect the impact of long-term exposure to shared risk factors, it might also point toward the co-occurrence of both conditions.

## Introduction

Cardiovascular diseases and cancer remain the leading causes of morbidity and mortality worldwide (1, 2). Several studies have shown that patients with cancer are at higher risk of developing cardiovascular disease (3, 4). This may be due to a cancer-induced hypercoagulable state (5), or as a consequence of the detrimental effects of cancer treatment on the vascular system (4, 6, 7). Additionally, it has also been proposed that atherosclerosis, the most important underlying condition of cardiovascular events, and cancer may share a common pathophysiology (8).

Common risk factors such as age, smoking, obesity, and genetic susceptibility are known to contribute to the risk of both atherosclerosis and cancer. Specific molecular pathways leading to atherosclerosis, including inflammation, oxidative stress, and uncontrolled cell proliferation, are also involved in the pathogenesis of cancer (9, 10). As such, both diseases are likely to co-occur. Although many studies have focused on the presence of atherosclerosis after cancer diagnosis, less is known about the presence and extent of atherosclerosis before cancer manifestation. This is of particular interest, since the first atherosclerotic lesions may already develop during infancy (11). Understanding the sequence and timing between these potentially interconnected diseases is pivotal as it may help to identify high-risk patients and to develop preventive strategies for both diseases.

Due to the central anatomical location in the arterial system, the presence and amount of atherosclerosis in the aortic arch may provide an easy measurable proxy of the systemic burden of atherosclerosis within an individual. As such, aortic arch atherosclerosis has repeatedly been linked to mortality, in particular also of non-cardiovascular origin, of which cancer represents a substantial part (12, 13). Hence, to further investigate the link between atherosclerosis and cancer, we determined the association between aortic arch calcification—as proxy for systemic atherosclerosis—with the subsequent risk of cancer within the setting of a large prospective population-based study.

## Method

### Setting

This study is embedded within the Rotterdam Study, a prospective population-based cohort study that investigates determinants and occurrence of chronic diseases in the middle-aged and elderly population. The design of the Rotterdam Study has been described in detail previously (14). At study entry, all participants were interviewed at home by a trained research assistant, followed by two visits to the research facility to undergo different examinations including laboratory assessments and imaging. Follow-up examinations take place every 3 to 5 years.

### Study Population

For the present study, we used the follow-up visit between 2003 and 2006 as baseline, because during this period, participants who visited the research center were invited to undergo non-contrast multi-detector computed tomography (MDCT) scanning of the aortic arch as part of a project of visualizing arterial calcification (15). We scanned 2,524 participants (response rate, 78%). Out of 2,524 scans, 6 scans were ungradable for aortic arch calcification because of the presence of image artifacts, leaving a total of 2,518 complete examinations with information on calcification. We excluded participants with a history of cancer (*n* = 114), resulting in 2,404 participants for analysis. The follow-up for cancer took place continuously and was completed for this study until January 1st, 2015.

The Rotterdam Study has been approved by the Medical Ethics Committee of the Erasmus MC (registration number MEC 02.1015) and by the Dutch Ministry of Health, Welfare and Sport (Population Screening Act WBO, license number 1071272-159521-PG). The Rotterdam Study has been entered into the Netherlands National Trial Register (NTR; www.trialregister.nl) and into the WHO International Clinical Trials Registry Platform (ICTRP; www.who.int/ictrp/network/primary/en/) under shared catalog number NTR6831. All participants provided written informed consent to participate in the study and to have their information obtained from treating physicians.

### Assessment of Aortic Arch Calcification

We used a 16-slice or 64-slice MDCT scanner (Somatom Sensation 16 or 64; Siemens, Forchheim, Germany) to perform non-contrast CT scanning. We scanned the aortic arch using an extra-cardiac scan that reached from the aortic arch to the intracranial vasculature (1 cm above the sella turcica). Detailed information on both scans is provided elsewhere (16, 17). As proxy of aortic arch atherosclerosis, we quantified aortic arch calcification on the extra-cardiac scan by including all calcification from the origin of the aortic arch (defined as the image in which the ascending and descending aorta merge into the inner curvature of the aortic arch) to the first 1 cm of the branches originating from the arch (18). Calcification volumes were calculated using dedicated software (Syngo Calcium Scoring; Siemens, Forchheim, Germany) and expressed in mm^3^.

### Assessment of Cancer

Diagnoses of cancer were based on medical records of general practitioners (including hospital discharge letters) and through linkage with Dutch Hospital Data, the Netherlands Cancer Registry, and histology and cytopathology registries in the region. Incident cancer was defined as any primary malignant tumor, excluding non-melanoma skin cancer. Only pathology-confirmed cancers were included in analysis to exclude the possibility of false-positive cancer diagnoses. Diagnoses were coded independently by two physicians according to the International Classification of Diseases, Tenth Revision (ICD-10). In case of discrepancy, consensus was sought through consultation with a physician specialized in internal medicine. Date of diagnosis was based on date of biopsy (solid tumors) and laboratory assessment (hematologic tumors), or—if unavailable—date of hospital admission or discharge letter. Follow-up of cancer registration was complete until January 1st, 2015.

### Measurement of Covariables

Information on educational level, smoking behavior, and use of antidiabetic, antihypertensive, lipid-lowering, and antithrombotic medication was obtained by trained interviewers. Educational level was classified into primary education, lower education (lower/intermediate general education or lower vocational education), intermediate education (intermediate vocational education or higher general education), or higher education (higher vocational education or university). Smoking status was categorized as never, current, or former. At the research center, height and weight were measured from which the body mass index (BMI; kg/m^2^) was computed. Furthermore, systolic and diastolic blood pressures were measured twice on the right arm with a random-zero sphygmomanometer of which the mean was used for analyses. Hypertension was defined as a systolic blood pressure of ≥140 mm Hg, a diastolic blood pressure of ≥90 mm Hg, or use of antihypertensive medication (19). Hypercholesterolemia was defined as use of lipid-lowering medication or serum total cholesterol >6.5 mmol/L. Diabetes mellitus was defined as use of antidiabetic medication, fasting serum glucose level ≥7.1 mmol/L, or random serum glucose level ≥11.1 mmol/L (20). We defined history of cardiovascular disease as history of myocardial infarction, stroke, percutaneous transluminal coronary angioplasty, and/or coronary artery bypass graft (21–23). Granulocyte count was measured using the COULTER® Ac·T diff2™ Hematology Analyzer (Beckman Coulter, San Diego, California, USA).

### Statistical Analysis

Considering the skewed distribution of aortic arch calcification volumes, we performed a natural log-transformation and added 1 mm^3^ to each non-transformed volume to deal with calcium scores of 0 (Ln[calcification volume + 1.00 mm^3^]). First, we used Cox proportional hazard models to determine the association between aortic arch calcification [per 1-standard deviation (SD) increase] and the subsequent risk of cancer. Model 1 was adjusted for age at MDCT scan and sex. To investigate to which extent any association would be driven by shared risk factors, model 2 was additionally adjusted for shared risk factors including educational level, smoking status, BMI, hypertension (24), hypercholesterolemia (25), diabetes mellitus, history of cardiovascular disease, and granulocyte count. We chose to use granulocyte count as markers of inflammation, since these blood cells in particular are related to larger volumes of arterial calcification (26).

Second, we computed tertiles of calcification severity and investigated associations with the risk of cancer, using the same two Cox proportional hazard models as described above and using the first tertile as reference category. As calcification volumes were larger in men than in women, the tertiles were computed sex-specifically. Also, considering differences in risk factors for atherosclerosis and cancer between men and women (27), we performed analyses stratified by sex.

For all Cox proportional hazard models, follow-up time was used as timescale and started at date of MDCT scan until date of incident cancer, death, loss to follow-up, or January 1st, 2015, whichever came first. Censoring participants at date of death allowed us to compute cause-specific hazard ratios (HRs). The proportional hazards assumption was met for all analyses (Schoenfeld residuals test, all *P* > 0.05).

To explore the robustness of our findings, we performed several sensitivity analyses to assess potential bias associated with mortality given the strong association between atherosclerosis and mortality (13). First, we restricted the analyses to shorter follow-up periods to limit the number of mortality events. To this end, participants with longer follow-up duration were censored at, respectively, 2, 3, 4, and 5 years after the MDCT scan. Second, we repeated the continuous analyses for the most common cancer types, i.e., breast cancer (among women), prostate cancer (among men), colorectal cancer, and lung cancer. Third, we stratified analyses by use of lipid-lowering and antithrombotic medication, and by median granulocyte count. In addition, we formally tested interaction by adding multiplicative interaction terms to the model.

To account for missing data of covariables (maximum amount of missing data: 5.9%), we used multiple imputation (*n* = five imputations) by chained equations along with age, sex, calcification volumes, cardiovascular risk factors, and cancer incidence. Statistical analyses were performed using STATA v.15 (StataCorp).

## Results

Characteristics of participants at time of MDCT scan are presented in Table 1. In addition, population characteristics stratified by tertiles of aortic arch calcification volumes are shown in Supplementary Table 1. The mean (SD) age was 69.5 years (6.8), and 52.5% were women. Among participants in the highest tertile of aortic arch calcification, a higher prevalence of cardiovascular risk factors was observed than in participants in the lowest tertile. During a median (interquartile range) follow-up of 9.6 years (8.9–10.5), 348 out of 2,404 participants were diagnosed with cancer, and 463 participants died. The most frequently diagnosed cancer types were prostate (39.2% among men), breast (34.4% among women), colorectal (16.1% overall), and lung (11.5% overall).

**Table 1:** Characteristics of the study population.

**Characteristic**	**All participants (*N* = 2,404)**	**Men (*N* = 1,143)**	**Women (*N* = 1,261)**
Age^*^	69.5 (6.8)	69.6 (6.6)	69.5 (6.9)
**Educational level**			
Primary	198 (8.2)	70 (6.1)	128 (10.2)
Lower	1023 (42.6)	298 (26.1)	725 (57.5)
Intermediate	737 (30.7)	444 (38.8)	293 (23.2)
Higher	446 (18.6)	331 (29.0)	115 (9.1)
Body mass index^*^	27.7 kg/m^2^ (4.1)	27.4 kg/m^2^ (3.5)	27.9 kg/m^2^ (4.5)
**Smoking**			
Never	687 (28.6)	157 (13.7)	530 (42.0)
Former	1339 (55.7)	777 (68.0)	562 (44.6)
Current	378 (15.7)	209 (18.3)	169 (13.4)
**Hypertension**			
No	604 (25.1)	283 (24.8)	321 (25.5)
Yes	1800 (74.9)	860 (75.2)	940 (74.5)
**Hypercholesterolemia**			
No	1387 (57.7)	721 (63.1)	666 (52.8)
Yes	1017 (42.3)	422 (36.9)	595 (47.2)
**Lipid-lowering medication use**			
No	1806 (75.1)	851 (74.5)	955 (75.7)
Yes	598 (24.9)	292 (25.5)	306 (24.3)
**Antithrombotic medication use**			
No	1813 (75.4)	794 (69.5)	1019 (80.8)
Yes	591 (24.6)	349 (30.5)	242 (19.2)
**Diabetes mellitus**			
No	2076 (86.4)	973 (85.1)	1103 (87.5)
Yes	328 (13.6)	170 (14.9)	158 (12.5)
**History of cardiovascular disease**			
No	2108 (87.7)	939 (82.2)	1169 (92.7)
Yes	296 (12.3)	204 (17.8)	92 (7.3)
Granulocyte count^†^	3.8 (3.0–4.6)	4.0 (3.2–4.8)	3.6 (2.9–4.4)
Aortic arch calcification^†^	263.8 mm^3^ (46.5–883.2)	296.9 mm^3^ (52.9–1009.5)	228.5 mm^3^ (41.8–825.0)

*Characteristics are measured at time of MDCT scan. Data are presented as frequency (percent) unless indicated otherwise. Numbers are shown after multiple imputation*.

* *Presented as mean (standard deviation)*.

*Presented as median (interquartile range)*.

We found no statistically significant association between continuous volumes of aortic arch calcification and the risk of cancer (Table 2). When investigating tertiles of calcification, we found that severe calcification was associated with a higher risk of cancer in the total population and in men (adjusted HR for the third tertile compared to the first tertile of aortic arch calcification in total population = 1.39, 95% CI = 1.04–1.86 and in men = 1.44, 95% CI = 1.00–2.09). This association was also observed in women, albeit not statistically significant (HR = 1.33, 95% CI = 0.83–2.13). Effect estimates were slightly attenuated when we corrected for different cardiovascular risk factors.

**Table 2:** The association between aortic arch calcification and the risk of cancer.

		**Risk of cancer**		
	**Aortic arch calcification**	***n*/*N***	**Model I HR (95% CI)**	**Model II HR (95% CI)**
		348/2,404		
Total population	Per 1-SD increase^*^		1.12 (0.99–1.27)	1.09 (0.96–1.24)
	T1	107/802	Ref.	Ref.
	T2	98/801	0.91 (0.69–1.20)	0.89 (0.67–1.18)
	T3	143/801	1.48 (1.12–1.95)	1.39 (1.04–1.86)
		131/1,261		
Women	Per 1-SD increase^*^		1.10 (0.90–1.33)	1.05 (0.88–1.28)
	T1	41/421	Ref.	Ref.
	T2	35/420	0.85 (0.54–1.34)	0.82 (0.52–1.30)
	T3	55/420	1.45 (0.93–2.27)	1.33 (0.83–2.13)
		217/1,143		
Men	Per 1-SD increase^*^		1.14 (0.97–1.33)	1.12 (0.96–1.32)
	T1	66/381	Ref.	Ref.
	T2	63/381	0.95 (0.67–1.35)	0.94 (0.66–1.34)
	T3	88/381	1.49 (1.04–2.12)	1.44 (1.00–2.09)

*Hazard ratios in model I are adjusted for age (and sex in the total population). Hazard ratios in model II are adjusted for covariates in model I plus adjustment for education, smoking status, body mass index, hypertension, hypercholesterolemia, diabetes mellitus, history of cardiovascular disease, and granulocyte count*.

*CI, confidence interval; HR, hazard ratio; n, number of participants with incident cancer; N, total number for participants; T, tertile*.

* *Ln(calcification volume + 1 mm^3^)–transformed volumes*.

When censoring participants with a follow-up duration longer than 2 years, we found that effect estimates were higher for both continuous aortic arch calcification volume as well as for severe calcification, albeit not statistically significant (adjusted HR per 1-SD increase in aortic calcification = 1.24, 95% CI = 0.90–1.71, and adjusted HR for the third tertile compared to the first tertile of aortic arch calcification = 1.53, 95% CI = 0.75–2.13). The effect estimates decreased with inclusion of longer follow-up duration (Table 3).

**Table 3:** The association between aortic arch calcification and the risk of cancer while censoring at different time points.

		**Risk of cancer**		
	**Aortic arch calcification**	***n*/*N***	**Model I HR (95% CI)**	**Model II HR (95% CI)**
		62/2,404		
Restricted to 2 years of follow-up	Per 1-SD increase^*^		1.25 (0.92–1.70)	1.24 (0.90–1.71)
	T1	16/802	Ref.	Ref.
	T2	19/801	1.15 (0.59–2.27)	1.13 (0.57–2.24)
	T3	27/801	1.59 (0.80–3.15)	1.53 (0.75–2.13)
		103/2,404		
Restricted to 3 years of follow-up	Per 1-SD increase^*^		1.13 (0.90–1.43)	1.12 (0.88–1.42)
	T1	28/802	Ref.	Ref.
	T2	30/801	1.02 (0.60–1.72)	1.01 (0.60–1.72)
	T3	45/801	1.45 (0.86–2.46)	1.43 (0.83–2.47)
		137/2,404		
Restricted to 4 years of follow-up	Per 1-SD increase^*^		1.04 (0.86–1.27)	1.04 (0.85–1.27)
	T1	44/802	Ref.	Ref.
	T2	39/801	0.83 (0.53–1.29)	0.82 (0.53–1.28)
	T3	54/801	1.08 (0.69–1.69)	1.06 (0.67–1.69)
		183/2,404		
Restricted to 5 years of follow-up	Per 1-SD increase^*^		1.02 (0.87–1.20)	1.02 (0.87–1.21)
	T1	61/802	Ref.	Ref.
	T2	51/801	0.80 (0.55–1.17)	0.81 (0.55–1.18)
	T3	71/801	1.11 (0.75–1.62)	1.11 (0.74–1.65)

*Hazard ratios in model I are adjusted for age (and sex in the total population). Hazard ratios in model II are adjusted for covariates in model I plus adjustment for education, smoking status, body mass index, hypertension, hypercholesterolemia, diabetes mellitus, history of cardiovascular disease, and granulocyte count*.

*CI, confidence interval; HR, hazard ratio; n, number of participants with incident cancer; N, total number for participants; T, tertile*.

* *Ln(calcification volume + 1 mm^3^)–transformed volumes*.

Regarding specific cancer types, we found that among women, aortic arch calcification was associated with a lower risk of breast cancer (HR per 1-SD increase in aortic arch calcification = 0.71, 95% CI = 0.52–0.98) and with a higher risk of lung cancer in the total population (HR = 2.35, 95% CI = 1.39–3.96, Table 4).

**Table 4:** The association between aortic arch calcification and the risk of different cancer types.

	HR per 1-SD increase in aortic arch calcification^*^		
	***n*/*N***	**Model I HR (95% CI)**	**Model II HR (95% CI)**
Breast cancer	45/1,261	0.75 (0.55–1.00)	0.71 (0.52–0.98)
Prostate cancer	72/1,143	1.08 (0.82–1.39)	1.07 (0.81–1.40)
Colorectal cancer	56/2,404	1.18 (0.86–1.63)	1.04 (0.76–1.43)
Lung cancer	40/2,404	2.87 (1.72–4.78)	2.35 (1.39–3.96)

*Hazard ratios in model I are adjusted for age (and sex in the total population). Hazard ratios in model II are adjusted for covariates in model I plus adjustment for education, smoking status, body mass index, hypertension, hypercholesterolemia, diabetes mellitus, history of cardiovascular disease, and granulocyte count*.

*CI, confidence interval; HR, hazard ratio; n, number of participants with incident cancer; N, total number for participants*.

* *Ln(calcification volume + 1 mm^3^)–transformed volumes*.

No effect modification was observed by lipid-lowering and antithrombotic medication use and by median granulocyte count. All interactions were tested on the multiplicative scale and did not reach statistical significance (*P* > 0.05).

## Discussion

In this population-based study, we found that only individuals with the most severe aortic arch calcification had a higher risk of cancer, in particular in the short term.

It has previously been shown that atherosclerosis and cardiovascular events occur after cancer diagnosis potentially as a result of cancer itself—by inducing a hypercoagulable state—and cancer treatment. Based on the shared risk factors and pathophysiology, we hypothesized that atherosclerosis is also associated with a subsequent higher risk of cancer and that the strength of this association would diminish after adjustment for shared risk factors. When investigating the association between the amount of aortic arch calcification and the risk of cancer, we found only a slightly higher risk, which was not statistically significant. However, when targeting the group of individuals in the highest tertile of calcification, we found 39% increase of the risk of cancer compared to those with the lowest tertile. Further investigation of this association demonstrated that the effect of atherosclerosis on cancer seems to be largest in the short term (during the first 2 years of follow-up). Although this indicates that severe atherosclerosis may be present before cancer diagnosis, it might also reflect reverse causation. It is possible that subclinical cancer development already influences the course of atherosclerosis. Since both atherosclerosis and cancer are conditions with a long preclinical phase, we cannot prove causality, nor can we rule out reverse causation.

Overall, we found no prominent differences in the association between atherosclerosis and cancer before and after adjustment for cardiovascular risk factors. This suggests that overall, traditional cardiovascular risk factors do not fully explain potential co-occurrence of atherosclerosis and cancer. This could point toward other factors, such as genetic variation or exogenous factors, that may explain the differential susceptibility to either atherosclerosis or cancer. However, exposure to shared risk factors might explain the higher risk of cancer in those individuals with the most severe aortic arch calcification and the strong association with lung cancer. It is likely that individuals who have been exposed to shared risk factors for a long period, and in high amounts, have both the largest volumes of aortic arch calcification and the highest risk of cancer. This suggests that the co-occurring deterioration of atherosclerosis and development of cancer is due to long and severe exposure to risk factors. However, also in these individuals, the size of the effect estimates only slightly diminished after correction for shared risk factors.

Sex differences—reflected by differences in hormonal levels—may also influence the apparent different impact of shared risk factors on the development of atherosclerosis and cancer. Aortic arch calcification was associated with a lower risk of breast cancer in women. It has previously been proposed that an inverse association between aortic atherosclerosis and cancer holds in particular for cancers that are hormone-dependent or highly affected by genetics rather than for those caused by exogenous factors (28). Also, aortic arch calcification in particular is strongly associated with mortality, indicating that the inverse relation between aortic arch calcification and cancer may partly be due to residual survival bias (13). More in-depth inquiry on this topic is required.

Several strengths of our study are worth mentioning. Our study is a large prospective population-based study and therefore less vulnerable to selection and information bias than retrospective ones. In addition, we have prospective and unbiased collection of many risk factors that are not available in healthcare databases. All cancers were pathology proven, which excludes the chance of misclassification. Also, we had an image-based assessment of calcification volumes and standardized ascertainment of cancer incidence.

Yet, some potential limitations need to be addressed. First, despite sufficient power to detect a large effect size of 1.5 for all cancers (α = 0.05, β = 0.80), we acknowledge the lack of statistical power to elaborate on specific cancer types. Second, strong associations of atherosclerosis with the risk of cardiovascular events and mortality may have weakened any potential association of atherosclerosis with cancer. Nevertheless, our finding that the most severe aortic calcification is associated with a higher risk of cancer—while these persons have the highest risk of mortality—might indicate that the effect of survival is limited. Third, the burden of atherosclerosis may influence the prognosis and course of cancer rather than the development itself. Ideally, measures of atherosclerosis at multiple time points are needed to also assess changes in atherosclerotic burden before cancer diagnosis. Future studies are needed to unravel differences in the etiology between atherosclerosis and cancer explained by other factors such as genetic and exogenous factors. Lastly, calcification is only a part of the atherosclerotic plaque. Non-calcified parts of the plaques cannot be visualized with non-enhanced CT. Nevertheless, it has been shown that calcification volume is an adequate measure for the total underlying plaque burden (29).

## Conclusions

We found that only individuals with the most severe aortic arch calcification had a higher risk of cancer, potentially through long-term parallel exposure to shared risk factors. Other factors, such as genetic variation or exogenous factors, may further explain susceptibility to either atherosclerosis or cancer.

## Data Availability Statement

Data can be obtained upon request. Requests shou towards the management team of the Rotterdam Study (secretariat.epi@erasmusmc.nl), which has a protocol for approving data requests. Because of restrictions based on privacy regulations and informed consent of the participants, data cannot be made freely available in a public repository.

## Ethics Statement

The studies involving human participants were reviewed and approved by the Medical Ethics Committee of the Erasmus MC (registration number MEC 02.1015) and by the Dutch Ministry of Health, Welfare and Sport (Population Screening Act WBO, license number 1071272-159521-PG). The Rotterdam Study has been entered into the Netherlands National Trial Register (NTR; www.trialregister.nl) and into the WHO International Clinical Trials Registry Platform (ICTRP; www.who.int/ictrp/network/primary/en/) under shared catalog number NTR6831. All participants provided written informed consent to participate in the study and to have their information obtained from treating physicians.

## Author Contributions

JT, KW, and DB contributed to conception and design of the research. JT, KW, RR, MV, BS, SS, MI, MK, and DB provided essential reagents or provided essential materials. JT and KW analyzed data. performed statistical analysis, and wrote the paper. JT, KW, and DB had primary responsibility for final content. JT, KW, RR, MV, BS, SS, MI, MK, and DB provided intellectual content to the paper. All authors have read and approved the final manuscript.

## Conflict of Interest

The authors declare that the research was conducted in the absence of any commercial or financial relationships that could be construed as a potential conflict of interest.
