# Supplementary material for: Aortic Arch Calcification and the Risk of Cancer: A Population-Based Cohort Study
Source: Front Oncol. 2020 Sep 11;10:1700. doi: 10.3389/fonc.2020.01700 (PMC7518099; doi:10.3389/fonc.2020.01700)
Supplement: Supplementary file 1 [file Table_1.docx]

Supplementary Material

**Table 1** Characteristics of the study population stratified by tertiles of aortic arch calcification volumes.

| Characteristic | Tertile 1 (N=802) | Tertile 2 (N=801) | Tertile 3 (N=801) |
| --- | --- | --- | --- |
| Age* | 66.1 (4.8) | 69.1 (6.0) | 73.4 (7.2) |
| Educational level |  |  |  |
| Primary | 32 (4.0) | 56 (7.0) | 110 (13.7) |
| Lower | 348 (43.4) | 329 (41.1) | 346 (43.2) |
| Intermediate | 237 (29.6) | 270 (33.7) | 230 (28.7) |
| Higher | 185 (23.1) | 146 (18.2) | 115 (14.4) |
| Body mass index* | 27.6 kg/m^2^ (3.9) | 27.8 kg/m^2^ (4.0) | 27.7 kg/m^2^ (4.3) |
| Smoking |  |  |  |
| Never | 279 (34.8) | 239 (29.8) | 169 (21.1) |
| Former | 425 (53.0) | 445 (55.6) | 469 (58.6) |
| Current | 98 (12.2) | 117 (14.6) | 163 (20.3) |
| Hypertension |  |  |  |
| No | 282 (35.2) | 202 (25.2) | 120 (15.0) |
| Yes | 520 (64.8) | 599 (74.8) | 681 (85.0) |
| Hypercholesterolemia |  |  |  |
| No | 518 (64.6) | 453 (56.6) | 416 (51.9) |
| Yes | 284 (35.4) | 348 (43.4) | 385 (48.1) |
| Lipid-lowering medication use |  |  |  |
| No | 662 (82.5) | 599 (74.8) | 545 (68.0) |
| Yes | 140 (17.5) | 202 (25.2) | 256 (32.0) |
| Antithrombotic medication use |  |  |  |
| No | 689 (85.9) | 618 (77.2) | 506 (63.2) |
| Yes | 113 (14.1) | 183 (22.8) | 295 (36.8) |
| Diabetes mellitus |  |  |  |
| No | 718 (89.5) | 696 (86.9) | 662 (82.6) |
| Yes | 84 (10.5) | 105 (13.1) | 139 (17.4) |
| History of cardiovascular disease |  |  |  |
| No | 757 (94.4) | 719 (89.8) | 632 (78.9) |
| Yes | 45 (5.6) | 82 (10.2) | 169 (21.1) |
| Granulocyte count^†^ | 3.6 (2.9-4.4) | 3.7 (3.0-4.6) | 4.0 (3.2-4.9) |
| Aortic arch calcification^†^ | 15.9 mm^3^ (0.6-46.6) | 263.9 mm^3^ (161.0-389.4) | 1356.2 mm^3^ (884.4-2491.9) |

Characteristics are measured at time of MDCT-scan. Data are presented as frequency (percent) unless indicated otherwise. Numbers are shown after multiple imputation.
* Presented as mean (standard deviation).

^†^ Presented as median (interquartile range).
